# Draft Genome Sequences of Sporulation-Impaired Bacillus pumilus Strain NRS576 and Its Native Plasmid p576

**DOI:** 10.1128/MRA.00089-19

**Published:** 2019-04-18

**Authors:** Jorge Val-Calvo, Andrés Miguel-Arribas, César Gago-Córdoba, Arancha López-Pérez, Gayetri Ramachandran, Praveen K. Singh, Ricardo Ramos-Ruiz, Wilfried J. J. Meijer

**Affiliations:** a Department of Virology and Microbiology, Centro de Biología Molecular “Severo Ochoa” (CSIC-UAM), Instituto de Biología Molecular “Eladio Viñuela” (CSIC), Universidad Autónoma Madrid, Madrid, Spain; b Genomics Unit Cantoblanco, Science Park, Madrid, Spain; University of Delaware

## Abstract

Bacillus pumilus spores can cause foodborne poisonings. *B. pumilus* strain NRS576 forms spores with a very reduced efficiency due to the presence of a plasmid, named p576. Here, we report the genome sequence of strain *B. pumilus* NRS576 and its plasmid p576.

## Abstract (precis)

Bacillus pumilus spores can cause foodborne poisonings. *B. pumilus* strain NRS576 forms spores with a very reduced efficiency due to the presence of a plasmid, named p576.

## ANNOUNCEMENT

Organisms in the *Bacillus* genus are Gram-positive bacteria that can form spores resistant to radiation, heat, and chemicals. Bacillus pumilus is present in soil samples, but some strains are associated with food poisoning, and spore formation is relevant to its pathogenicity (1–3). *B. pumilus* NRS576 forms spores with low efficiency due to a plasmid (4), p576, which we have sequenced previously (5). Here, we report the chromosomal and plasmid sequences of strain NRS576 obtained from the *Bacillus* Genetic Stock Center. A total DNA sample (6), obtained from cells growing in LB medium at 37°C, was used for sequence determination with the Illumina MiSeq platform. DNA libraries were prepared with the NEBNext DNA library prep kit for Illumina (New England Biolabs). Briefly, 1 µg DNA was sonicated, and fragments of ∼675 or 1,075 bp were selected. DNA ends were repaired, A-tailed, and ligated to adapters. Next, fragments were PCR amplified (8 cycles) and quality checked on a DNA 7500 chip on a 2100 Bioanalyzer (Agilent). Library sizes of ∼800 and 1,200 bp were isolated, validated (Bioanalyzer), and titrated with quantitative PCR (qPCR). After denaturation, the libraries were seeded on a flow cell (MiSeq v2, 2 × 150 bp) at a density of 16 pM. The sequencing rendered 3,963,212 (800-bp library) and 2,015,074 (1,200-bp library) paired-end reads. Data processing was done with default parameters. Adapters (Cutadapt [7]) and low-quality sequences (Sickle 1.33 [8]) were removed. After verifying the quality of the processed data (FastQC [9]), *de novo* assembly was performed (SPAdes v3.9.1 [10–13]). Three apparent extrachromosomal elements were detected with plasmidSPAdes (13), (i) plasmid p576, (ii) bacteriophage phiX174 (added as a control for amplification and sequencing [14]), and (iii) sequences similar to part of the Brevibacillus laterosporus DSM25 *bogC* gene cluster. PhiX174 sequences and contigs smaller than 250 bp were removed, and sequences similar to *bogC* were considered genomic DNA. General statistics obtained with the bioinformatic tool Quast (15) gave an *N*_50_ value of 313,965 bp and an *L*_50_ value of 5.

One 43,328-bp contig corresponded to p576, which is 106 bp smaller than previously determined (5). The plasmid p576 contains direct-repeat sequences. The apparent deletions correspond to these repeats. Otherwise, the p576 sequences differ in one single base pair (gene *64* codon 492 [GAA462GGA]). We sequenced p576 and genomic DNA with mean coverages of about 70 and 30, respectively, and this implies that p576 has a copy number of two. The previously published p576 nomenclature (5) was respected. Sanger (5) and next-generation sequencing (NGS)-determined p576 sequences are publicly available.

NRS576 genomic sequences were located on 32 contigs with a total length of 3,675,031 bp and 41.6% GC content. Based on our annotation with PROKKA (16), the genome contains 3,811 putative genes, distributed as 3,641 coding DNA sequences (CDS), 86 noncoding RNAs (ncRNA), 73 tRNAs, 10 rRNAs, and 1 transfer-messenger RNA (tmRNA).

### Data availability.

This whole-genome shotgun project has been deposited at DDBJ/ENA/GenBank under accession number UWJF00000000. The version described here is the first version. Raw sequence reads have been submitted to the Sequence Read Archive (SRA) (accession numbers ERR2811649 and ERR2811650, corresponding to 2 × 150-bp paired-end sequences of 800- and 1,200-bp fragments, respectively). The p576 sequences are available under DDBJ/ENA/GenBank accession numbers LR026976 (Sanger) and LR026977 (NGS).

## References

[1] Suominen I, Andersson MA, Andersson MC, Hallaksela A-M, Kampfer P, Rainey FA, Salkinoja-Salonen M 2001 Toxic *Bacillus pumilus* from indoor air, recycled paper pulp, Norway spruce, food poisoning outbreaks and clinical samples. Syst Appl Microbiol 24:267–276. doi:10.1078/0723-2020-00025. 11518331

[2] Nieminen T, Rintaluoma N, Andersson M, Taimisto A-M, Ali-Vehmas T, Seppälä A, Priha O, Salkinoja-Salonen M 2007 Toxinogenic *Bacillus pumilus* and *Bacillus licheniformis* from mastitic milk. Vet Microbiol 124:329–339. doi:10.1016/j.vetmic.2007.05.015. 17611049

[3] Logan NA 2012 Bacillus and relatives in foodborne illness. J Appl Microbiol 112:417–429. doi:10.1111/j.1365-2672.2011.05204.x. 22121830

[4] Lovett PS 1973 Plasmid in *Bacillus pumilus* and the enhanced sporulation of plasmid-negative variants. J Bacteriol 115:291–298. 4717517 10.1128/jb.115.1.291-298.1973 PMC246242

[5] Singh PK, Ballestero-Beltrán S, Ramachandran G, Meijer WJJ 2010 Complete nucleotide sequence and determination of the replication region of the sporulation inhibiting plasmid p576 from *Bacillus pumilus* NRS576. Res Microbiol 161:772–782. doi:10.1016/j.resmic.2010.07.007. 20863889

[6] Sambrook J, Fritsch EF, Maniatis T 1989 Molecular cloning: a laboratory manual. Cold Spring Harbor Laboratory Press, Cold Spring Harbor, New York.

[7] Martin M 2011 Cutadapt removes adapter sequences from high-throughput sequencing reads. EMBnet J 17:10–12. doi:10.14806/ej.17.1.200.

[8] Joshi NA, Fass JN 2011 Sickle—a sliding-window, adaptive, quality-based trimming tool for FASTQ files. https://github.com/najoshi/sickle.

[9] Andrews SA 2011 A quality control tool for high throughput sequence data. https://www.bioinformatics.babraham.ac.uk/projects/fastqc.

[10] Bankevich A, Nurk S, Antipov D, Gurevich AA, Dvorkin M, Kulikov AS, Lesin VM, Nikolenko SI, Pham S, Prjibelski AD, Pyshkin AV, Sirotkin AV, Vyahhi N, Tesler G, Alekseyev MA, Pevzner PA 2012 SPAdes: a new genome assembly algorithm and its applications to single-cell sequencing. J Comput Biol 19:455–477. doi:10.1089/cmb.2012.0021. 22506599 PMC3342519

[11] Nurk S, Bankevich A, Antipov D, Gurevich AA, Korobeynikov A, Lapidus A, Prjibelski AD, Pyshkin A, Sirotkin A, Sirotkin Y, Stepanauskas R, Clingenpeel SR, Woyke T, McLean JS, Lasken R, Tesler G, Alekseyev MA, Pevzner PA 2013 Assembling single-cell genomes and mini-metagenomes from chimeric MDA products. J Comput Biol 20:714–737. doi:10.1089/cmb.2013.0084. 24093227 PMC3791033

[12] Prjibelski AD, Vasilinetc I, Bankevich A, Gurevich A, Krivosheeva T, Nurk S, Pham S, Korobeynikov A, Lapidus A, Pevzner PA 2014 ExSPAnder: a universal repeat resolver for DNA fragment assembly. Bioinformatics 30:i293–i301. doi:10.1093/bioinformatics/btu266. 24931996 PMC4058921

[13] Antipov D, Hartwick N, Shen M, Raiko M, Lapidus A, Pevzner PA 2016 plasmidSPAdes: assembling plasmids from whole genome sequencing data. Bioinformatics 32:3380–3387. doi:10.1093/bioinformatics/btw493. 27466620

[14] Mukherjee S, Huntemann M, Ivanova N, Kyrpides NC, Pati A 2015 Large-scale contamination of microbial isolate genomes by Illumina PhiX control. Stand Genomic Sci 10:18. doi:10.1186/1944-3277-10-18. 26203331 PMC4511556

[15] Gurevich A, Saveliev V, Vyahhi N, Tesler G 2013 QUAST: quality assessment tool for genome assemblies. Bioinformatics 29:1072–1075. doi:10.1093/bioinformatics/btt086. 23422339 PMC3624806

[16] Seemann T 2014 Prokka: rapid prokaryotic genome annotation. Bioinformatics 30:2068–2069. doi:10.1093/bioinformatics/btu153. 24642063

